# A latent profile analysis of relationship satisfaction and self‐regulation among low‐income participants who attended relationship education with a partner

**DOI:** 10.1111/famp.13051

**Published:** 2024-09-06

**Authors:** Ryan G. Carlson, Naomi J. Wheeler, Xun Liu, Nakita Carroll

**Affiliations:** ^1^ Department of Educational and Developmental Science University of South Carolina Columbia South Carolina USA; ^2^ Department of Counseling and Special Education Virginia Commonwealth University Richmond Virginia USA; ^3^ Foundations of Education Virginia Commonwealth University Richmond Virginia USA; ^4^ Department of Psychiatry and Behavioral Science Mercer University Macon Georgia USA

**Keywords:** couple distress, latent profile analysis, relationship education, relationship satisfaction, relationship self‐regulation

## Abstract

Relationship education has shown promising effects for low‐income couples on outcomes such as promoting positive communication, improving global relationship satisfaction, parenting, and individual psychological distress. Studies also indicate that couples' baseline distress (e.g., relational and individual) moderates outcomes. Yet, few studies implemented a person‐centered approach to analyzing data for those who participate in relationship education. In a sample of 488 low‐income opposite‐gendered couples, we identified latent profile groups for men and women based on self‐reported relationship satisfaction and behavioral self‐regulation scores, thus incorporating both relational and individual factors. Results yielded a three‐class solution for men and a four‐class solution for women. We then examined group profile differences in individual psychological distress and relationship satisfaction change scores after completing the relationship education intervention (12 h of PREP's *Within Our Reach*). Results indicated significant differences, suggesting that group membership can predict overall improvements in both psychological and relationship distress. Thus, RE programmers and policymakers may consider flexible delivery (e.g., more or less content; more or less intense coaching) that considers overall baseline relational and/or individual functioning as opposed to a one‐size‐fits‐all method.

Relationship education (RE) consists of skills‐based workshops designed to teach couples about effective communication, healthy conflict resolution, and related relational skills (Stanley et al., 2020). RE has emerged as a promising intervention for low‐income couples since government funding began in the early 2000s to support implementation and evaluation (Hawkins et al., 2022). Low‐income couples experience unique relationship stressors and do not have access to relationship support interventions. Relationship education is accessible, reduces the stigma of relationship help‐seeking given the focus on skill‐attainment, and is designed to help low‐income couples develop and maintain healthy and stable relationships. Despite some inconsistency in effects from RE for global relationship satisfaction and stability (Johnson & Bradbury, 2015), meta‐analytic studies (i.e., Arnold & Beelman, 2018; Hawkins et al., 2022; Hawkins & Erickson, 2015), as well as several experimental and quasi‐experimental evaluations, have demonstrated positive changes in communication, dyadic coping, relationship satisfaction, relationship skills, conflict, emotion regulation, individual distress, and parenting (Adler‐Baeder et al., 2013, 2022; Barden et al., 2022; Carlson et al., 2014; Rhoades, 2015; Roddy et al., 2020) among participants who attended RE either in person or online. Additionally, some studies showed that couples with higher baseline distress (individual or relational) experienced greater overall improvement (Amato, 2014; Carlson et al., 2017; Petch et al., 2012; Roddy et al., 2020; Williamson et al., 2016), challenging a prior conceptualization of RE as a preventive intervention. Relationship education is often considered a primary prevention intervention for couples who experience fewer overall relationship problems. As such, RE is delivered universally for all who attend, despite differences in baseline functioning. However, more research is needed to understand the characteristics of couples who attend relationship education, as well as how those characteristics influence changes over the course of the intervention (Markman & Rhoades, 2012). Therefore, the primary goal of this study was to identify profiles of men and women (i.e., clusters of individuals with a similar pattern of responses) who enrolled in relationship education as a couple and compare pre‐post change differences among profile groups. The study aimed to (a) use self‐report intake data on relationship satisfaction and relationship maintenance strategies (i.e., behavioral relationship self‐regulation) to develop profile groups, and (b) use the profiles developed to test differences of pre‐post change scores on relationship satisfaction and individual psychological functioning to examine profile validity.

## RELATIONSHIP EDUCATION AND COUPLE DISTRESS

As a skills‐based, manualized relationship enhancement intervention, relationship education began as an early prevention approach for couples anticipating future relationship challenges (Ooms & Wilson, 2004). There are numerous RE curricula that have been developed since its inception, but the overall aim of relationship education is for couples to learn about effective communication and healthy conflict resolution skills (Larson, 2004). Specific RE curricula vary, in that they include unique features based on the theory supporting their development. For instance, cognitive and behavior therapy principles underlie many RE curricula. As a result, RE curricula often emphasize relationship skill development to bolster changes in relationship strategies and effort to maintain and sustain the relationship, otherwise known as *behavioral relationship self‐regulation* (RSR; Halford et al., 1994). RSR is a dyadic process influential to perceived relationship satisfaction (Halford et al., 2007; Hardy et al., 2015) and partner attachment and communication quality (Knapp et al., 2015). Relationship self‐regulation has been positively correlated with improvements in relationship quality (Rackham & Larson, 2020). However, relationship self‐regulation has not been assessed within the context of relationship education skills programs. Given the emphasis on skill attainment in relationship education, relationship self‐regulation seems an important construct to consider.

After nearly 20 years of government‐funded evaluations of RE with diverse and low‐income populations, evidence supports the notion that many RE participants attend because they are already experiencing significant relationship distress and do not otherwise have access to relationship interventions (Hawkins et al., 2022). Such couples often demonstrate greater gains than their less‐distressed counterparts (Amato, 2014; Bradford et al., 2016; Petch et al., 2012; Williamson et al., 2016). Couples experiencing greater distress may demonstrate more improvement because they have more room to grow (i.e., the ceiling effect). However, many of these same distressed, and under‐resourced, couples experience significant challenges in attending consecutive RE workshops (e.g., transportation, childcare, shift‐style work schedules; Carlson et al., 2014; Williamson et al., 2019). Thus, special attention should be considered to the unique challenges facing low‐income couples who experience greater relationship distress (Williamson et al., 2019).

Online relationship education has emerged as a recent efficacious approach to addressing some contextual challenges (Doss et al., 2016, 2020). Yet, couples participating in online RE also experience stressors and may be more likely to seek relationship support than their more traditional in‐person RE‐attending counterparts (Doss et al., 2016). One recent randomized controlled trial with 742 low‐income couples found that couples attending online RE reported both mental health and behavioral health effects (*d* ranged from 0.11–0.42 on the total sample), and that baseline distress moderated several of these effects (*d* ranged from 0.16–0.45 for couples with initial distress; Roddy et al., 2020). Williamson et al. (2019) compared distress levels between low and higher‐income couples who enrolled in online RE. Results indicated that lower‐income couples reported less relationship satisfaction, less relationship stability, and more contextual stressors (such as children living at home, lack of full‐time employment, and worse perceived health) than their higher‐income counterparts.

Considering distressed couples' improvements, as well as the increasing numbers of distressed couples attending RE (e.g., Hawkins et al., 2017), some scholars called for changes to the universal nature of RE implementation (Bradford et al., 2015). Bradford and colleagues suggested a CRE+ model that differentiates between high and low‐distress couples and applies a therapeutic component, as well as improved screening (hence the + in CRE+), to address the unique needs of high‐distress couples. However, not everyone agrees with a tailored approach to RE. Markman and Ritchie (2015) noted a concern about taking a clinical approach to RE implementation. They argued that utilizing a clinical model of RE with couples makes RE less accessible and increases the stigma associated with the approach. Accessibility and reduced stigma are two likely reasons couples may choose RE over clinical counseling. Hawkins (2019) proposed a tiered approach to RE implementation that serves as a potential middle‐ground. The first three tiers include relationship literacy education, micro‐interventions, and more traditionally implemented programmatic RE, respectively. The fourth tier represents clinically delivered education that is tailored and delivered in one‐on‐one counseling. Hawkins' approach is conceptual in nature but underscores the current perspective that distress level likely influences RE effectiveness and should be considered during service delivery. While there is no clear ‘right’ answer, the debate is noteworthy. It does not change the fact that RE practitioners will be providing services to couples who are already experiencing relationship problems and/or to those who are experiencing heightened anxiety/depression, thus potentially impacting their ability to engage in relationship self‐regulation (e.g., effort). As such, RE researchers and practitioners would benefit from understanding more about distress levels for RE participants and how distress might moderate outcomes.

A recent meta‐analysis of RE programs funded by the Administration for Children and Families (ACF) conducted by Hawkins et al. (2022) found that later programs in the ACF's initiative had stronger outcomes than its earlier programs. Hawkins and colleagues suggested that improved program evaluation considers best practices, like for whom and how the intervention works best, has led to stronger outcomes in these later RE programs (Hawkins et al., 2022). Despite the advances in RE implementation science, specifically, studies demonstrating distress differences among participants, few published studies have identified profile groups. One study developed outcomes‐focused clusters from baseline measures on relationship skills (Dupree et al., 2016). Dupree and colleagues found three clusters: the first represented those who began RE rating themselves lower on relationship skills but reporting modest gains at post‐assessment; the second cluster rated themselves the lowest on relationship skills and reported the largest post‐intervention gains; and the third cluster rated themselves in the middle range on baseline relationship skills, and reported moderate post‐intervention gains. These clusters help describe the experiences of RE participants. However, more information is needed to understand the association of baseline functioning with post‐intervention functioning.

## THE PRESENT STUDY

The present evaluation is influenced by previous studies indicating the bi‐directional relationship between couple relationship functioning and individual functioning (Baucom et al., 1998; Isakson et al., 2006). For example, the marital discord model of depression (Beach, 1991) posits that couple relationship interventions can be used to treat individual mental health (e.g., depression) of partners while also addressing relational issues. Some RE studies have examined the individual benefits of attending relationship education (e.g., Roddy et al., 2020), but to our knowledge, none have sought to identify latent profiles based on these factors. Thus, the purpose of the present study was to identify meaningful latent profiles of overall relationship satisfaction and relationship self‐regulation of couple relationships among low‐income, ethnically diverse individuals, based on their self‐reported ratings of relationship satisfaction and relationship self‐regulation.

### Data analytic strategy

An exploratory approach, latent profile analysis (LPA) was taken to analyze the number of latent classes, and no hypotheses about the specific number of latent classes resulting from the analyses were made. After identifying latent classes, we investigated the validity of these latent profiles by examining differences in the pre‐post change score for individual psychological functioning measured by the Outcomes Questionnaire 45.2 (OQ45.2; Lambert et al., 2004) and on relationship functioning measured by the Relationship Assessment Scale (RAS; Hendrick, 1998) between groups. Because women tend to report more distress in relationships compared to men (Jackson et al., 2014), and women often initiate relationship help‐seeking (Doss et al., 2003), we anticipated that there would be distinct distress profiles for men and women and thus analyzed their data separately. We hypothesized that men and women in classes with overall lower levels of relationship satisfaction and relationship self‐regulation would report more psychological and relational functioning gains than men and women in other groups. The study was guided by the following research question: After establishing participant profiles based on relationship satisfaction and relationship self‐regulation, are there differences between group membership profiles on pre‐post change for individual psychological and relationship distress before and after attending relationship education?

## METHOD

### Participants

We extracted data from 488 heterosexual couples (*N* = 976 individuals) to comprise the current sample (i.e., a small number of same‐sex couples were removed for the purposes of this analysis to align with theoretical and empirical distinguishability by gender). All couples enrolled to participate in community‐based relationship education with Prevention and Relationship Education's (PREP) *Within Our Reach* (WOR; *n* = 296; 61%) or the *Within Our Reach Plus* (WOR Plus – an adaptation that included one additional week of career skills; *n* = 192; 39%) program during the fourth year of Project TOGETHER, a federally‐funded relationship education initiative located in a large city in the Southeastern region of the United States. Project staff recruited participants from community events, as well as partnering community agencies, such as the Women, Infants, and Children Program and local county health departments. Several participants learned about the project through word‐of‐mouth, flyers posted throughout the community, or local advertisements. Study inclusion criteria required that participants be (a) at least 18 years of age, (b) in a committed relationship, and (c) voluntarily interested in attending at least 12‐h of relationship education with their committed partner (i.e., the WOR Plus included 15 h of RE). Recruitment staff contacted the interested individuals telephonically to review program details and determine eligibility as defined by the inclusion criteria.

Participants included primarily Hispanic couples with low incomes. Options for race and ethnicity were predetermined in accordance with federal reporting and included Hispanic (yes or no) and racial categories for either American Indian/Alaska Native, Asian, Black/African American, Native Hawaiian/Other Pacific Islander, White, or Other. The open response for other races resulted in many participants indicating a country (e.g., Colombia, Honduras) or territory (e.g., Puerto Rico). Because we analyzed data separately for men and women, we present the following demographics separately as well. Men reported an average monthly income of $1995.56 (SD = $2268.89), with a modal number of 2 children. Men ranged in age from 19 to 80 years (*M* = 37.09, SD = 12.26), and the majority reported their ethnicity as Hispanic (52%; *n* = 256), followed by White (24%, *n* = 117), African American (17%, *n* = 81), and Other (7%, *n* = 34). Women reported an average monthly income of $984.80 (SD = $1194.11), with a modal number of two children. Women ranged in age from 18 to 74 years (*M* = 35.02, SD = 11.52), and reported their ethnicity as Hispanic (57%, *n* = 277); White (21%, *n* = 105); African American (17%; *n* = 81), or Other (5%; *n* = 25). The majority of couples reported being married (64%), followed by those in a committed relationship (30%), and engaged (6%).

### Procedures and intervention

Prior to beginning the study and collecting data, the University of Central Florida IRB approved all study procedures. After confirming eligibility and availability, staff scheduled couples to attend the intake appointment only after speaking with both members of the couple. The enrollment and intake occurred in a group format on the first night of the RE workshop, just prior to beginning the curriculum.

Enrolled couples received PREP's WOR curriculum (Stanley et al., 2008). PREP is widely used and recognized as an evidenced‐based couple and relationship education intervention (Jakubowski et al., 2004). WOR's lesson topics included: three keys to successful relationships, communication danger signs and time out, the speaker‐listener technique, events, issues and hidden issues, expectations, problem‐solving, love styles, personality, fun and friendship, and commitment matters. These skills address effective communication strategies using structured techniques for couples to practice together. Participants received the content in one of three workshop formats: (a) four consecutive weeknights for 3 h each meeting, (b) four consecutive Saturdays for 3 h each meeting, or (c) three consecutive Saturdays for 4 h each meeting. A male–female dyad co‐facilitated workshops that included a combination of lectures, videos, and group and couple activities. Facilitators represented a range of demographic backgrounds representative of the population (e.g., race, ethnicity, age, education, and relationship status). Facilitators received PREP training, participated in regularly scheduled supervision meetings, and received peer reviews to help ensure curriculum fidelity. More information on facilitator training and access to curricular content can be found at prepinc.com. The program offered PREP workshops in English or Spanish, depending on couples' preferences. Roughly half the workshops were facilitated in each language. Once a couple joined a workshop group, the majority of couples attended the entirety of the curriculum in the same multi‐couple educational group. Some couples received what the local project termed *WOR* Plus, which referenced one additional week added to the workshops that addressed job and career readiness skills. Couples enrolled in the Plus version only if they reported being unemployed or under‐employed and identified an interest in participating in career skills training prior to enrollment.

The project considered participants enrolled once the couple completed the group intake and pre‐assessments. Assessment measures were available in English and Spanish, and most assessments had pre‐existing translated versions for use. For the researcher‐developed instruments, Spanish‐translated assessments were cross‐validated by the research and project team members who represented diverse Spanish‐speaking cultures. Once enrolled, participants received a number of incentives and program services to support retention and engagement. Incentives to participate included childcare and meals provided at all workshops and gift cards distributed incrementally (e.g., couple members received $25 US dollars at the first workshop session, third session or 75% completion, and fourth session or 100% completion) as participants achieved program benchmarks up to $75 per participant for full completion of the PREP curriculum.

### Instruments

Although individuals completed several questionnaires as part of their participation in Project TOGETHER, we include only those pertinent to the current analysis.

#### Demographic questionnaire

The *Adult History Intake Questionnaire* is a researcher‐developed measure completed by all program participants in order to collect data on participant demographics, contact information, relationship status, educational attainment, employment status, monthly income, and military status. We also used information from the intake questionnaire to identify couples' current needs (e.g., employment assistance, transportation assistance, childcare, and other community referrals), as well as community resources that might benefit them. For the current study, the intake questionnaire was completed by each individual participant, and data was used to describe participants.

#### Relationship satisfaction

We used the Relationship Assessment Scale (RAS; Hendrick, 1998), a seven‐item measure, to assess overall relationship satisfaction. Participants responded to items on a Likert scale from 1 to 5. Scale anchors varied by item. Mean scores were calculated by summing the responses (items four and seven are reverse coded) and then dividing by seven. According to Hendrick (1998), a score of 4.0 or higher indicates greater relationship satisfaction, while average scores closer to 3.5 indicate relationship distress for men, and average scores ranging from 3.0 to 3.5 for women indicate relationship distress and overall dissatisfaction. The RAS has been used reliably with low‐income couples in previous studies (e.g., Carlson et al., 2017). In the current study, the reliability of RAS scores at baseline in the male and female groups was 0.89 and 0.91. At the post‐test, the reliability of RAS scores in the male and female groups was 0.87 and 0.91, respectively. The baseline RAS score and pre‐post change score of RAS were used in the current study.

#### Individual distress

The Outcomes Questionnaire 45.2 (OQ; Lambert et al., 2004) is a 45‐item measure of overall psychological distress and includes a total score, as well as three sub‐scales: symptom distress, social role, and interpersonal relationships. Participants responded to the 45 items on a Likert‐type scale ranging from *never* (0) to *almost always* (*4*), and the measure was scored on a 0–180 scale, with a higher total score indicating a greater level of overall distress and a low score indicating less distress. Example items include: *I feel tired; I feel weak; I am a happy person; and I feel lonely*. Clinically distressed individuals score above 63 on the OQ total score and 14 or more may be interpreted as meaningful change in total distress. The authors also indicate cutoff scores and indicators for reliable change on each subscale: symptom distress cutoff, 36, change of 10 or more; interpersonal relationship distress cutoff, 15, change of eight or more; and social role performance cutoff, 12, change of seven or more. The OQ internal consistency scores range from 0.71 to 0.93 and test–retest reliabilities range between 0.66 and 0.82 (Lambert et al., 2004). The OQ has been used in previous studies with low‐income participants (e.g., Carlson et al., 2014, 2017). In this study, the overall alpha reliabilities of OQ scores for male and female groups were both 0.92 at baseline. The overall alpha reliabilities of OQ score for men and women were 0.92 and 0.93, respectively at post‐test. We also calculated a pre‐post change in OQ scores.

#### Relationship self‐regulation

The Behavioral Self‐Regulation for Effective Relationships Scale: Self‐Report (BSRERS‐Self) was used to assess participants' relationship self‐regulation. The BSRERS (Wilson et al., 2005) is a 16‐item measure with two forms: self‐report (BSRERS‐Self) and partner‐report (BSRERS‐Partner). We used the BSRERS‐Self in the present analysis. For each item in BSRERS‐Self, participants rated the extent to which the statement is true of their own behavior in the relationship on a 5‐point scale from 1 (*not true at all*) to 5 (*very true*). The 16‐item BSRERS has a two‐factor structure of relationship self‐regulation strategies and effort, and each factor has high internal consistency (Wilson et al., 2005). In this study, we only used the baseline BSRERS‐Self score to contribute to participant profile development. The overall reliabilities of BSRERS‐Self score for men and women were 0.83 and 0.85, respectively. A higher total BSRERS‐Self score indicates a greater level of overall positive relationship regulation, and a lower score indicates less or ineffective regulation.

Attrition varied by measure, gender, and time point. Men were missing 4.7% and 34.9% of OQ scores at baseline and post‐assessment, respectively. Additionally, men were missing 4.3% and 20% of RAS scores at baseline and post‐assessment. Women were missing 4.3% and 34.3% of OQ scores and 4.3% and 19.4% of RAS scores at baseline and post‐assessment, respectively. For the baseline BSRERS‐self scores, the missing percentages were below 5% for both men and women. For all measures, missing data at baseline was due to item non‐response, while the missing data at post‐assessment was due to study attrition. To address potential bias due to attrition, we estimated via full information maximum likelihood (FIML) in Mplus. As an extension of maximum likelihood, FIML takes advantage of all possible data points in the analysis.

### Data analyses

We used a Latent Profile Analysis (LPA) to identify groups with different configurations of relationship satisfaction and relationship self‐regulation for men and women from baseline scores. LPA identifies groups of individuals who are similar to each other and differentiates them from other groups (Magidson & Vermunt, 2002; Muthén & Muthén, 2017). Mplus *v*.7 was used to conduct LPA, and Maximum Likelihood estimation with Robust standard error (MLR), the default setting in Mplus, was adopted as the maximum likelihood estimator. Due to the exploratory nature of LPA, the number of latent profiles is not known beforehand (Orpinas et al., 2015). Thus, we compared models with increasing numbers of latent classes (starting from one class) to obtain a model that achieved the number of classes fitting the data well.

In the present study, we used the RAS and BSRERS‐Self as latent class indicators and examined a series of models with one to four classes. To achieve the most appropriate model, we compared statistical fit indices, including the Akaike Information Criterion (AIC), Bayesian Information Criterion (BIC), sample‐size adjusted BIC (aBIC), the Lo–Mendell–Rubin likelihood ratio test (LMRT), and Bootstrapped Likelihood ratio test (BLRT). Additionally, we examined entropy values. According to Ramaswamy et al. (1993), entropy values that range from 0 to 1 and values 0.80 or more indicate good separation and distinction between the identified groups as well as greater classification accuracy. No single criterion is able to determine the best solution. Models need to be compared based on interpretability, theory, and statistical criteria (Marsh et al., 2009). Another important criterion in determining the best model is to satisfy the parsimony (i.e., adequately accounting for the complexity of the data using the fewest latent classes) of the model (Orpinas et al., 2015).

## RESULTS

### Model selection for men

To select the most appropriate model, we tested the fit statistics of the latent profiles from a one‐class solution up to a four‐class solution. Table one presents the fit indices for each solution. According to Ramaswamy et al. (1993), for the AIC, BIC, and aBIC, lower values indicate a better fit. AIC and BIC values for men decreased across the four solutions, while the BIC value at the three‐ and four‐class solutions remained fairly consistent. We obtained significant values for the LMRT and BLRT results, which support the test model over a model with one fewer class (G‐1; Ramaswamy et al., 1993). The *p*‐value of LMRT was 0.07, which was no more significant at the four‐class level with the alpha level set at 0.05. Entropy values steadily increased from the two‐class model to the four‐class model for men (i.e., entropy at the three‐class level 0.72 and 0.76 at the four‐class level). We considered all the fit indices and determined the three‐class solution provided the most informative interpretation of the classes, each of which had clear characteristics. See Table 1 for indicator means by class.

**TABLE 1:** Fit indices for one to four‐class models for men.

Model	AIC	BIC	aBIC	LMR value	LMR *p* value	BLRT value	BLRT *p* value	Entropy
1‐Class Model	4794.873	4811.63	4798.94	N/A	N/A	N/A	N/A	N/A
2‐Class Model	4693.142	4722.47	4700.26	102.23	0.0001	107.731	<0.0001	0.694
3‐Class Model	4654.001	4695.90	4664.16	42.84	0.01	45.141	<0.0001	0.719
4‐Class Model	4641.315	4695.79	4654.53	17.73	0.07	18.686	<0.0001	0.761

Abbreviations: aBIC, sample size adjusted BIC; AIC, Akaike Information Criterion; BIC, Bayesian Information Criterion; BLRT, Bootstrap Likelihood Ratio Test; LMR, Lo–Mendell–Rubin Test.

### Class descriptions for men

#### Class 1: Highly satisfied and self‐regulated class

The men in the first class (*n* = 243; 49.8%) had a high degree of relationship satisfaction (*M* = 4.46, SE = 0.05) and relationship self‐regulation (*M* = 60.81, SE = 0.99). Specifically, this class had consistently higher scores in relationship satisfaction than the other two classes on the RAS scale, and consistently higher scores in relationship self‐regulation than the other two classes on the BSRERS‐self scale. Thus, we named this group the *highly satisfied and self‐regulated group*.

#### Class 2: Moderately satisfied and self‐regulated class

The men in the second class (*n* = 198; 40.57%) were satisfied in their couple relationship and self‐regulated relationally, to a medium degree, reporting moderate relationship satisfaction scores (*M* = 3.56, SE = 0.10) as compared to the other two classes reflected on the RAS scale. The men in this class also reported a moderate degree of self‐regulated behaviors in terms of relationship self‐regulation strategies and effort (*M* = 52.61, SE = 0.85) when compared with the other two classes reflected on the BSRERS scale. We named this group the *moderately satisfied and self‐regulated group*.

#### Class 3: Unsatisfied and low self‐regulated class

The men in this class were not satisfied in their couple relationships and reported less relationship self‐regulation (*n* = 47; 9.63%). Specifically, this class had consistently lower scores for relationship satisfaction (*M* = 2.37, SE = 0.15) than the other two classes on the RAS scale. This class also had consistently lower scores for relationship self‐regulation strategies and effort (*M* = 46.02, SE = 1.72) in comparison with the other two classes on the BSRERS scale. We named this group the *unsatisfied and low self‐regulated group*.

### Between class differences for men

After we identified the three classes using LPA with RAS and BSRERS‐Self baseline scores as predictors, the Auxiliary option (bch) in M*plus* (Bakk & Vermunt, 2016) was performed to determine whether the change in mean OQ scores (i.e., OQ‐symptom distress, OQ‐interpersonal relations, OQ‐social role performance, and OQ‐total) and RAS differed across the latent classes (see Table 2).

**TABLE 2:** Latent profile means and standard deviations for OQ‐SD, OQ‐IR, OQ‐SR, OQ‐T with men.

Variable	Unsatisfied and low self‐regulated		Moderately satisfied and self‐regulated		Highly satisfied and self‐regulated		Omnibus test		Class comparisons
	(U; *n* = 47)		(S; *n* = 198)		(H; *n* = 243)				
	1		3		2				
	*M*	SD	*M*	SD	*M*	SD	*χ* ^2^	*p*	
OQ‐SD‐change	4.24	2.53	5.81	0.95	2.50	0.83	5.67	0.06	S > H (*p* = 0.02)
OQ‐IR‐change	2.41	0.89	3.06	0.52	1.09	0.48	6.75	0.03	S > H (*p* = 0.01)
OQ‐SR‐change	2.94	0.94	1.47	0.41	0.79	0.36	5.38	0.07	U > H (*p* = 0.03)
OQ‐T‐change	9.60	3.62	10.33	1.55	4.37	1.38	7.53	0.02	S > H (*p* = 0.01)
RAS‐change	0.57	0.12	0.36	0.05	−0.08	0.03	79.75	<0.001	U > H (*p* < 0.001) S > H (*p* < 0.001)

Abbreviations: OQ‐IR, Outcomes Questionnaire 45.2 Subscale: Interpersonal Relations; OQ‐SD, Outcomes Questionnaire 45.2 Subscale: Symptom Distress; OQ‐SR, Outcomes Questionnaire 45.2 Subscale: Social Role Performance; OQ‐T, Outcomes Questionnaire 45.2 Total.

The Highly Satisfied Class remained lowest on OQ‐symptom distress change score, OQ‐interpersonal relationships change score, OQ‐social role performance change score, OQ‐total change score, and RAS‐average change score. All the change scores from the Highly Satisfied Class were significantly lower than the change scores from either the Unsatisfied (OQ‐SR‐change score) or Moderately Satisfied Class (OQ‐SD‐change score; OQ‐IR‐change score; OQ‐T‐change score), or both (RAS change score), which indicates that the least relationally satisfied and relationally regulated men experienced greater changes in distress.

### Model selection for women

To select the most appropriate model, we tested latent profiles from a one‐ to five‐class solution and examined the fit statistics of each (see Table 3). The values of AIC, BIC, and aBIC in the Female Group decreased across model iterations until the four‐class model. Values of AIC, BIC, and aBIC were larger in the five‐class model when compared to the four‐class model. The *p*‐value of the LMRT was 0.41, which was no more significant at the five‐class level with the alpha level set at 0.05. Thus, the fit indices supported the four‐class solution for the Female Group. Entropy values in the Female Group steadily increased from the two‐class model to the four‐class model and dropped at the five‐class model. Considering statistical findings and the interpretability of the results based on theory, we accepted the four‐class model as a highly informative model for understanding the current latent profiles and the model with clearly distinguishable characteristics (see Table 4 for indicator means by class).

**TABLE 3:** Fit indices for one to five‐class models for women.

Model	AIC	BIC	aBIC	LMR value	LMR *p* value	BLRT value	BLRT *p* value	Entropy
1‐Class Model	4965.173	4981.94	4969.24	N/A	N/A	N/A	N/A	N/A
2‐Class Model	4876.384	4905.72	4883.50	89.95	<0.0001	94.8	<0.0001	0.56
3‐Class Model	4829.994	4871.90	4840.16	49.71	0.0003	52.4	<0.0001	0.76
4‐Class Model	4813.008	4867.48	4826.22	21.81	0.0067	23.0	<0.0001	0.79
5‐Class Model	4813.469	4880.51	4829.73	5.26	0.4128	5.5	0.4286	0.71

Abbreviations: aBIC, sample size adjusted BIC; AIC, Akaike Information Criterion; BIC, Bayesian Information Criterion; BLRT, Bootstrap Likelihood Ratio Test; LMR, Lo–Mendell–Rubin Test.

**TABLE 4:** Latent profile means and standard deviations for OQ‐SD, OQ‐IR, OQ‐SR, OQ‐T for four‐class profile solution with women.

Variable	Highly unsatisfied and low self‐regulated		Unsatisfied and low self‐regulated		Moderately satisfied and self‐regulated		Highly satisfied and self‐regulated		Omnibus overall test		Class comparisons
	(HU; *n* = 40)		(U; *n* = 102)		(S; *n* = 184)		(HS; *n* = 162)				
	*M*	SD	*M*	SD	*M*	SD	*M*	SD	*χ* ^2^	*p*	
OQ‐SD‐change	5.64	2.88	6.23	1.6	6.54	1.28	3.38	0.64	7.41	0.06	HS < S (*p* = 0.04)
OQ‐IR‐change	2.60	1.03	3.22	0.87	4.15	0.54	0.81	0.47	22.36	<0.001	HS < U (*p* = 0.01); HS < S (*p* < 0.001)
OQ‐SR‐change	0.34	0.93	1.62	0.58	1.93	0.51	0.98	0.38	3.58	0.31	None
OQ‐T‐change	8.58	4.11	11.07	2.63	12.63	1.96	5.16	1.13	13.55	0.004	HS < U (*p* = 0.04); HS < S (*p* = 0.002)
RAS‐change	0.37	0.15	0.39	0.07	0.39	0.05	−0.08	0.03	90.68	<0.001	HS < U (*p* < 0.001); HS < S (*p* < 0.001); HS < HU (*p* = 0.004)

Abbreviations: OQ‐IR, Outcomes Questionnaire 45.2 Subscale: Interpersonal Relations; OQ‐SD, Outcomes Questionnaire 45.2 Subscale: Symptom Distress; OQ‐SR, Outcomes Questionnaire 45.2 Subscale: Social Role Performance; OQ‐T, Outcomes Questionnaire 45.2 Total.

### Class descriptions for women

#### Class 1: Highly satisfied and self‐regulated class

The individuals in this class had a high degree of relationship satisfaction and relationship self‐regulation (*n* = 162; 33.2%). Specifically, this class had consistently higher scores in relationship satisfaction (*M* = 4.59, SE = 0.03) than the other three classes on the RAS scale and had consistently higher scores in relationship self‐regulation (*M* = 64.85, SE = 0.82) than the other three classes on the BSRERS‐Self scale. Thus, we named this group the *highly satisfied and self‐regulated group*.

#### Class 2: Moderately satisfied and self‐regulated class

The individuals in this class are also satisfied in couple relationships and self‐regulated behaviorally (*n* = 184; 37.7%), with lower scores than Class 1. Specifically, this class had consistently higher satisfaction in the couple relationship (*M* = 3.67, SE = 0.05) than Class 3 and Class 4 as measured by the RAS. Class 2 also had consistently higher relationship self‐regulation strategies and effort (*M* = 54.38, SE = 0.83) than Class 3 and Class 4 as measured by the BSRERS scale. We name this group the *satisfied and self‐regulated group*.

#### Class 3: Unsatisfied and low self‐regulated class

The individuals in this class were not satisfied in their couple relationships and were less self‐regulated (*n* = 102; 20.9%). Specifically, this class had consistently lower scores in relationship satisfaction (*M* = 2.88, SE = 0.08) than Class 1 and Class 2 on the RAS scale. This class also had consistently lower scores in self‐regulated behaviors in terms of relationship self‐regulation strategies and effort (*M* = 52.78, SE = 1.22) than Class 1 and Class 2 on the BSRERS scale. We named this group the *unsatisfied and low self‐regulated group*.

#### Class 4: Highly unsatisfied and low self‐regulated class

The individuals in this class were not satisfied in their couple relationships and were less self‐regulated (*n* = 40; 8.2%). Specifically, this class had the lowest scores in relationship satisfaction (*M* = 1.93, SE = 0.10) than the other three classes on the RAS scale. Class four also had the lowest score in relationship self‐regulation strategies and effort (*M* = 52.28, SE = 2.28) in comparison to BSRERS scores with the other three classes. We named this group the *highly unsatisfied and low self‐regulated group*.

### Between class differences for women

After we identified the four classes using LPA with RAS and BSRERS‐Self baseline scores as predictors, we conducted the Auxiliary option (bch) in M*plus* (Bakk & Vermunt, 2016) to determine whether the change of mean scores of OQ‐45 (e.g., OQ‐symptom distress, OQ‐ interpersonal relationships, OQ‐social role performance, and OQ‐total) and RAS differed across the latent classes (see Table 4).

The Highly Satisfied Class remained lowest on OQ‐symptom distress change score, OQ‐interpersonal relationships change score, OQ‐total change score, and RAS‐average change score. All the change scores from the Highly Satisfied Class, except for the OQ‐social role change score, were significantly lower than the change scores from either the Highly Unsatisfied, Unsatisfied, or Moderately Satisfied Class (OQ‐SD‐change score) or both Unsatisfied and Moderately Satisfied Class (OQ‐IR‐change score; OQ‐T‐change score), or all three of them (RAS‐change score). Thus, the results showed that the Highly Satisfied Class gained the least from the RE intervention in terms of improvements in relationship satisfaction and psychological distress compared to either the Highly Unsatisfied, Unsatisfied, or Moderately Satisfied Classes.

## DISCUSSION

Study results indicated that both men and women can be classified into groups based on their baseline levels of relationship satisfaction and relationship self‐regulation. The initial predictors correlated such that, as relationship satisfaction declined, so too did a person's report of their effort and strategies for relationship maintenance (i.e., relationship self‐regulation). RE historically focuses on the development of relationship skills and strategies; thus, our results add to existing knowledge of factors that influence psychological and relational outcomes among participants in RE. Findings are particularly noteworthy given that the present study is one of the first to identify RE participant profiles from dynamic relationship variables and with a non‐White majority sample of low‐income couples. Additionally, the latent groups moderated change as both men and women in the highest satisfaction‐regulation groups experienced significantly less change in individual psychological distress and relationship satisfaction than their less satisfied‐regulated counterparts. Results are consistent with previous findings that people with higher levels of relationship distress experience a more significant change in functioning in relationship education (Amato, 2014; Carlson et al., 2017; Roddy et al., 2020) and add the effect of pre‐existing behaviors/strategies used to maintain the relationship (i.e., relationship self‐regulation). Additionally, as evidence continues to demonstrate that RE is not solely a primary prevention, more research should be conducted on specific factors that moderate or mediate change for distressed couples (e.g., pre‐existing characteristics that can help determine a more tailored treatment approach). Low‐income couples who attend relationship education programs may not be attending to prevent future problems but instead address existing relationship problems. Thus, experimental studies should be conducted that randomly assign participants based on baseline distress group. Such research would provide a more rigorous test of participant distress. Policy implications should only be considered after more rigorous testing. For example, if highly distressed couples continue to demonstrate positive effects from relationship education, it may be an efficient use of federal monies to emphasize support for distressed couples' participation in programming.

### Determination of baseline profiles and participant gender

We classified profiles of men and women attending RE as a couple by their baseline relationship satisfaction and self‐regulation. As a result, we concluded that the data best fit as a three‐class solution for men and a four‐class solution for women. Overall, both men and women included profiles for (a) highly satisfied and self‐regulated, (b) moderately satisfied and self‐regulated, and (c) unsatisfied and low self‐regulated profiles. Additionally, women also included a fourth profile for *highly* unsatisfied and low self‐regulated (about 8% of women). The majority of participants fit the categorization for the moderate to high satisfaction and self‐regulation classes, replicating prior findings that the majority of couples, regardless of socioeconomic risk, were relatively stable and satisfied in their relationships (Williamson et al., 2019).

Fewer participants seemed to be unsatisfied and low self‐regulated, yet the low satisfaction‐regulation groups accounted for about 10% of men and 30% of women at baseline (when unsatisfied and highly unsatisfied female groups were combined). Furthermore, more women reported higher levels of relational distress than men at baseline. As a community‐based sample, we would have predicted no differences in satisfaction by gender (Jackson et al., 2014); however, higher percentages of dissatisfied women are similar to gender‐based satisfaction differences found in clinical samples (Jackson et al., 2014) and may suggest parallels to initiation of couples counseling, where women often drive a couple's initial engagement in clinical services (Doss et al., 2003). For couples with higher socio‐demographic risk, perhaps RE provides a more accessible method of relationship intervention when distress levels are high. About one in three women enrolled in RE with high baseline relational distress, consistent with assertions that framing RE as primary prevention may not accurately depict the realities of the couples who enroll – in particular, for women who enroll with higher distress and dissatisfaction in the relationship. Prior studies identified gender differences in relationship satisfaction, where women tended to be less relationally satisfied when navigating periods of higher stress (Rusu et al., 2020). Given that most of the sample reported low income, our findings may further add to our understanding of the role of family stress rooted in economic precarity for relational stability (Conger et al., 1990). Women who enroll in RE with higher baseline relationship dissatisfaction also showed a slower rate of improvement than their male counterparts after attending RE (Liu et al., 2020). The least satisfied partner (in this case, women) may initiate RE attendance, but they may not experience the same benefits as their more satisfied partner.

### Baseline profile as a moderator of change

The second goal of the current study was to identify under which circumstances of relational satisfaction and self‐regulation couples were most amendable to change. Similar to subsequent research, overall participants in RE reported pre to post‐improvements in relational and psychological distress (Adler‐Baeder et al., 2013, 2022; Arnold & Beelman, 2018; Carlson et al., 2014; Hawkins & Erickson, 2015; Rhoades, 2015). Additionally, men and women with moderate to high baseline relational distress seemed to benefit the most from RE in terms of their reported change in psychological and relational distress (e.g., Amato, 2014; Bradford et al., 2016; Carlson et al., 2017; Petch et al., 2012; Williamson et al., 2016). Results of the current study are additive to the existing literature on differential outcomes from RE in that our profiles included dynamic variables (satisfaction, relationship self‐regulation), which are more amenable to change across the course of a relationship than fixed sociodemographic variables often associated with baseline risk. Therefore, risk profiles based on relational and psychological distress may be helpful for practitioners who aim to support the varied needs and goals of couples and families who seek out relational support.

Comparison of profile differences in change also showed that for men and women, the most distressed and most satisfied couples changed the least because of the RE intervention. The potential exists for ceiling effects among the higher satisfied‐regulated groups and also underscores the need to better understand what other kinds of benefit, beyond satisfaction or relationship self‐regulation, these kinds of couples and individuals may experience given the persistence of sociodemographic forms of risk. From our anecdotal experience with couples in RE, perhaps for highly satisfied‐regulated couples, RE may serve to support the maintenance of positive relationship choices, affirm relationship values, or develop ties between group members as a form of social support amidst contextual threats to relationship stability often reported by low‐income couples (Halpern‐Meekin, 2019). More research is needed to identify the motivations to attend and outcomes experienced by baseline satisfied‐regulated men and women, which may differ from those with greater presenting distress.

Conversely, compared to the higher satisfaction‐regulation profiles, moderate profiles for men and women showed the largest gains in psychological and relational distress. Specifically, moderate profiles for men and women yielded larger gains for relationship satisfaction, total distress, and subscales for interpersonal relationship and symptom distress. In fact, although none of the male or female classes indicated a mean change interpretable as meeting the clinically meaningful threshold, the moderate groups came the closest. Female mean OQ total change for the moderately satisfied and self‐regulated class approached clinical significance (OQ total change *M* = 12.62, SE = 1.96; meaningful change defined as a score change of 14 or more (Lambert et al., 2004)), followed by the unsatisfied and low self‐regulated class mean change in total distress (*M* = 11.07, SE = 2.63). Similarly, men in the moderately satisfied and self‐regulated class indicated mean change (*M* = 10.33, SE = 1.55), where the margin of error (at 95% confidence) and range for actual mean total distress change include clinically meaningful values for the moderate male and female groups. Thus, men and women in the moderate distress profiles saw the largest improvements in psychological distress that also approached clinical thresholds for improvement. In contrast, the highest distressed groups improved, but not to the same degree, where additional support to achieve clinically meaningful change may be needed.

Prior studies of RE interventions have demonstrated positive associations with individual distress outcomes (Bradford et al., 2014; Carlson et al., 2017). In this case, results suggest that baseline relationship dynamics differentially influence gains in individual distress such that couples benefit most psychologically when relationship distress is moderate to low (rather than extremely low or high relationship distress). Interestingly, the lowest satisfied‐regulated profiles (i.e., U for men, HU for women) showed no significant differences in change in psychological distress when compared to the other profile classes. So, although they experienced improvements in distress, their reported benefits from participation did not exceed those of other couples that started with higher or lower baseline distress. Relationship and individual functioning are correlated (McGill & Adler‐Baeder, 2019); therefore, this finding may reflect ceiling effects for the most relationally content and may also reflect the powerful influence of relationship distress on individual psychological health, including gains from intervention.

The only exception to the lack of significant difference in profile change was that for men in the unsatisfied‐low regulated group and changes in social role distress. Work and family stress exert bidirectional influence on one another, and gender or personal orientation to gender roles moderates these relationships (Zhao et al., 2019). Thus, it is possible that relationally distressed men may uniquely benefit from RE in terms of the spillover of stress/contentment between work and their relationship with their partner. Past research established the intersectional influence of race, ethnicity, and gender on work‐to‐family and family‐to‐work conflict (Ammons et al., 2017), where improvements to relationship satisfaction may have distinct effects on stress associated with work among men. Thus, improvements reported by men in the most distressed group are overall similar to gains achieved by the other profiles and are more pronounced for work‐related stress.

Differences in relational and psychological distress change related to baseline satisfaction and relationship self‐regulation align with recommendations for initial screening and tailored approaches to RE implementation (Bradford et al., 2015; Stanley et al., 2020). In our study, the men and women with the highest and lowest baseline distress did not benefit to the same degree as individuals with moderate to low distress. Therefore, high and low‐distressed couples present with unique needs that a universal approach may not adequately address. Our results may provide initial support for a more tailored approach to the delivery of RE in line with Hawkins' (2019) recommendations. Couples who report high levels of satisfaction and relationship self‐regulation at intake may benefit from RE in ways not currently captured through typical outcome measures such as changes to relationship distress or satisfaction. Future research should aim to explore highly satisfied couples experience of RE to identify other relational mechanisms that may be positively influenced through participation in terms of outcomes such as hope (Hawkins et al., 2017), development of social support (Carlson et al., 2020; Halpern‐Meekin, 2019), or affirmation/maintenance of relationship values and expectations. Couples who present with moderate satisfaction or high dissatisfaction may need something more, including other forms of relationship intervention (Stanley et al., 2020), in addition to the manualized RE, to address their psychological and relational distress. Lastly, RE researchers may need to further examine factors that influence presenting levels of distress and explore the efficacy of tiered approaches to intervention.

### Limitations

This study has limitations that need to be considered. First, we analyzed the data from heterosexual couples only and for men and women separately. While this process revealed separate groups for men and women, we cannot draw conclusions about couples, or between couple functioning. Further, the profiles and associations found in the present study need to be examined in more diverse partnerships including same‐sex couples or to account for the gender identity of participants. Second, we did not include a control group and therefore cannot infer any causality regarding the effectiveness of the intervention. Using change scores also inhibits the interpretation of causal effects and may add the potential for bias in the results. Future research should aim to include a more rigorous evaluation of the magnitude of outcome change by baseline characteristics of a couple. Third, we included a group of participants from an adapted version of PREP and used a non‐probability sampling strategy. We did not account for the slight curriculum variance in our analysis and cannot attribute any changes to this adaptation. Finally, sampling bias may influence our results, and members of the general population may differ from couples who voluntarily participated in the RE program.

## CONCLUSION

Overall, results reveal differences in baseline characteristics of men and women with low‐income and predominately Hispanic backgrounds related to perceived relationship satisfaction and self‐regulation. Profiles were associated with differences in change in psychological and relational distress after participation in RE. Results support the potential utility of screening couples upon enrollment for baseline functioning to inform tailored RE intervention.
